# Identification of General and Heart-Specific miRNAs in Sheep (*Ovis aries*)

**DOI:** 10.1371/journal.pone.0143313

**Published:** 2015-11-24

**Authors:** Alessandro Laganà, Dario Veneziano, Tyler Spata, Richard Tang, Hua Zhu, Peter J. Mohler, Ahmet Kilic

**Affiliations:** 1 Department of Molecular Virology, Immunology and Medical Genetics, Comprehensive Cancer Center, The Ohio State University, Columbus, OH, United States of America; 2 Department of Clinical and Molecular Biomedicine, University of Catania, Catania, Italy; 3 Department of Surgery, The Ohio State University, Columbus, OH, United States of America; 4 The Davis Heart and Lung Research Institute, Departments of Physiology & Cell Biology and Internal Medicine, The Ohio State University Medical Center, Columbus, OH, United States of America; Federico II University of Naples, ITALY

## Abstract

MicroRNAs (miRNAs or miRs) are small regulatory RNAs crucial for modulation of signaling pathways in multiple organs. While the link between miRNAs and heart disease has grown more readily apparent over the past three years, these data are primarily limited to small animal models or cell-based systems. Here, we performed a high-throughput RNA sequencing (RNAseq) analysis of left ventricle and other tissue from a pre-clinical ovine model. We identified 172 novel miRNA precursors encoding a total of 264 mature miRNAs. Notably, 84 precursors were detected in both the left ventricle and other tissues. However, 10 precursors, encoding 11 mature sequences, were specific to the left ventricle. Moreover, the total 168 novel miRNA precursors included 22 non-conserved ovine-specific sequences. Our data identify and characterize novel miRNAs in the left ventricle of sheep, providing fundamental new information for our understanding of protein regulation in heart and other tissues.

## Introduction

Coronary artery disease is the most common cause for mortality and development of heart failure in the United States [1–4]. Large animal models have been developed to study myocardial infarction with resultant ischemic heart failure [5–12]. The ovine model is particularly enticing in the study of heart failure due to its lack of collateral circulation, the resistance to fatal arrhythmias, and its anatomical similarities to humans [6,9,10,13]. However, the molecular mechanisms underlying development of heart failure in this critical pre-clinical model are unknown. In fact, we lack even a basic fundamental knowledge of the molecular pathways related to transcriptional and translational regulation in this species.

miRNAs are small non-coding RNAs that act as post-transcriptional regulators of gene expression [14,15]. Their mature form (≅18÷22 nt long) is incorporated into a protein complex called RISC (RNA-induced silencing complex) to which they confer binding specificity to target mRNA molecules. miRNAs can bind their targets through partial or perfect complementarity and inhibit their translation or promote their degradation. Target recognition is often mediated by the seed region at the 5' end of the mature miRNA, although a supplementary or compensatory role of the central and 3' end of the miRNA has been observed [16].

miRNAs play a crucial role in many biological processes and their dysregulation is linked to a variety of diseases with significant impact in important cellular pathways [17–20]. Recently, miRNAs have been linked with heart failure, albeit primarily in a descriptive manner [21–23]. Several works have reported the discovery of ovine miRNAs and the latest version of miRBase (Release 21, June 2014) contains 106 precursors and 153 mature miRNAs [24–30]. However, there remains a paucity of data on the miRNAs in sheep cardiac tissue.

The role of miRNAs in both diagnosis and treatment of heart failure remains enticing. Therefore, we defined and characterized novel miRNAs in the left ventricle of *Ovis aries* through high-throughput RNA sequencing (RNA-Seq) and a multi-step computational approach. Our results provide novel foundational work for understanding cardiac regulation in pre-clinical models by contributing to the characterization of the sheep “miRNAome”.

## Materials and Methods

All studies were conducted with approval by the Institutional Animal Care and Use Committee (IACUC) at The Ohio State University (Study number: 2012A00000040). Adult male Dorset sheep weighing between 50–70 kg were used for this study (n = 5). Strict adherence was kept to the Guide for the Care and Use of Laboratory Animals of the National Institutes of Health.

### Tissue Harvesting and RNA isolation

All animals were sedated, and all attempts were made to minimize animal discomfort as previously described [31]. The animals underwent a 5th intercostal space left thoracotomy, and an expeditious cardiectomy was performed. Autologous platelets from sheep’s own blood were pre-spun to form platelet aggregates. Then, employing selective cardiac catheterization, these were injected into the left anterior descending artery to create a reproducible area of myocardial infarction. This did not necessarily produce ischemic heart failure as that is a chronic model (which requires weeks) and we were measuring the acute effects (3 days) of a myocardial infarction [5].

In addition, a laparotomy and craniotomy were performed with procurement of lung, liver, kidney, spleen, pancreas and brain tissue.

The euthanization procedure strictly followed the Ohio State University IACUC approved method of euthanasia for vertebrate animals. In brief, the sheep were anesthetized using telazol 4–10 mg/kg and Isoflurane and intubated. After confirming the animals were under deep anesthesia (no response to deep tissue stimulus, lack of jaw tone, decreased respiration) the chest was opened to access the heart. The major vessels were cut to facilitate exsanguination and the heart and tissues removed for further analysis.

The tissue was separated into two groups with the test group being left ventricular (LV) tissue and the control group being all others (Global Library). The tissue collected was immediately flash frozen in liquid nitrogen and stored in -80 C. RNA isolation was performed using the mirVana PARIS isolation kit (Ambion, Austin, Texas). RNA integrity was confirmed with agarose gel electrophoresis stained with ethidium bromide with a 28S:18S ratio ≥ 2.0 as confirmation of RNA integrity. The purity of RNA was confirmed via UV spectroscopy using A260 and A280 ratio.

### Reverse transcription (RT) and preamplification

Reverse transcription and pre-amplification were performed according to the manufacturer’s instructions using TaqMan MicroRNA RT kit, Megaplex RT Primers, and Megaplex PreAmp Primers (Life Technologies Corporation, Carlsbad, CA). The mixture of total RNA with Megaplex RT primers, RNase Inhibitor and MultiScribe Reverse Transcriptase was reverse transcribed utilizing a C1000 thermal Cycler (Bio-Rad Laboratories Incorporated, Hercules, CA).

### Illumina Deep Sequencing

Two small RNA libraries were generated from LV and global samples using the Illumina Truseq^TM^ Small RNA Preparation kit according to Illumina’s TruSeq^TM^ Small RNA Sample Preparation Guide [REF1]. The purified cDNA library was used for cluster generation on Illumina’s Cluster Station and then sequenced on Illumina GAIIx following vendor’s instruction for running the instrument. Raw sequencing reads (40 nts) were obtained using Illumina’s Sequencing Control Studio software version 2.8 (SCS v2.8) following real-time sequencing image analysis and base-calling by Illumina's Real-Time Analysis version 1.8.70 (RTA v1.8.70). The extracted sequencing reads were stored in file separate raw data files and were then used in the standard data analysis, which is described in the Results and in the Bioinformatics Methods sections.

### Quantitative Stem-Loop Real-Time PCR

The expression of selected mature miRNAs in the LV sample was confirmed by Stem-Loop qRT-PCR performed with a TaqMan Human MicroRNA A array (version 2.0; Life Technologies Corporation, Carlsbad, CA) in association with TaqMan Universal Master Mix II (Life Technologies Corporation, Carlsbad, CA). The threshold cycle (Ct) = the fractional cycle number above a given threshold with normalization to U6 snRNA. The mean Ct of each sample was calculated as ΔCt with analysis via a commercially available software (SDS Relative Quantification Software, Applied Biosystems, Life Technologies Corporation, Carlsbad, CA). The relative quantification was solved with the equation of 2^-ΔCt^. The snRNA U6 was used as internal control. All microRNAs were considered expressed if Ct values were < 35. The conserved miRNAs were validated using TaqMan Assays for *Ovis aries*, where available, or 100% homologous miRNAs from other species (e.g. *Bos taurus* and *Homo sapiens*). The non-conserved miRNAs were validated using custom designed TaqMan Assays.

### Bioinformatics Analysis

For each sample, raw sequence reads were extracted from image data and then processed by a proprietary pipeline script, ACGT101-miR v4.2 (LC Sciences), which employed a series of filter to remove various un-mappable sequencing reads [32,33]. Identical reads were clustered into unique families and stored in FastQ files, annotated with their copy numbers. In the subsequent filtering step, low-quality sequences due to sample preparation, sequencing chemistry and processes, and the optical digital resolution of the sequencer detector were removed. The remaining sequences, with lengths between 15 and 32 bases, were grouped by families of unique sequences and stored in FastQ files as mappable reads.

The actual miRNA detection was carried out by applying miRDeep2, a software pipeline for the identification of miRNAs from deep sequencing data (https://www.mdc-berlin.de/rajewsky/miRDeep) [34]. miRDeep2 consists of a series of scripts which makes use of other software pieces such as the short read aligner Bowtie and the RNA secondary structure prediction tool RNA fold from the Vienna RNA package [35,36]. Briefly, the short reads are aligned to the genome, then all candidates whose structure and read signature are inconsistent with Drosha/Dicer processing are filtered out. Potential stem-loop precursors are assigned a score according to a Bayesian probabilistic model of miRNA biogenesis, which uses information such as the presence of a miRNA passenger strand, the presence of a conserved miRNA seed, and the absolute and relative energetic stability of the hairpin.

The mappable reads FastQ files from LV and the global library were processed by miRDeep2 using the *Ovis aries* v3.1 genome reference sequence downloaded from NCBI (ftp://ftp.ncbi.nlm.nih.gov/genomes).

For the prediction of novel miRNAs, we set a score cut-off threshold of 5, as it was the lowest score cut-off that yielded a signal-to-noise ratio higher than 10:1 (37:1), similarly to what has been described in other papers [37–40]. All the candidates with score below 5 were discarded. We didn’t employ the same filter for the detection of known miRNAs present in miRBase, as we considered their precursors already validated, but we filtered out all those candidates, novel and known, with mature read count below 10, this being a reasonable and commonly used count threshold [41–43].

All the candidates were processed by the miRBase, NCBI and RFam implementations of BLAST (miRBase: http://www.mirbase.org/search.shtml, NCBI: http://blast.ncbi.nlm.nih.gov/Blast.cgi, RFam: http://rfam.sanger.ac.uk/search), in order to assess their evolutionary conservation and filter out candidates that matched potential repetitive sequences and other types of short RNAs. The remaining candidates were further analyzed by employing ad-hoc scripts developed in the Ruby language (https://www.ruby-lang.org), in order to compute basic descriptive statistics about their genomic distribution, clustering and isomiRs, classify them into families, group multiple precursors encoding the same mature sequences and evaluate the dominant precursor arm in the different samples. The candidate mature miRNAs were processed by the software miRiam, in order to predict potential targets from the UCSC database of 3’ UTR sequences of the sheep (http://genome.ucsc.edu/) [44]. The lists of predicted targets were submitted to the online tool Ingenuity Pathway Analyzer (IPA), which we used to carry out the functional analysis.

## Results and Discussion

### Generation of RNA libraries from sheep and processing of sequencing data

**Fig 1:**
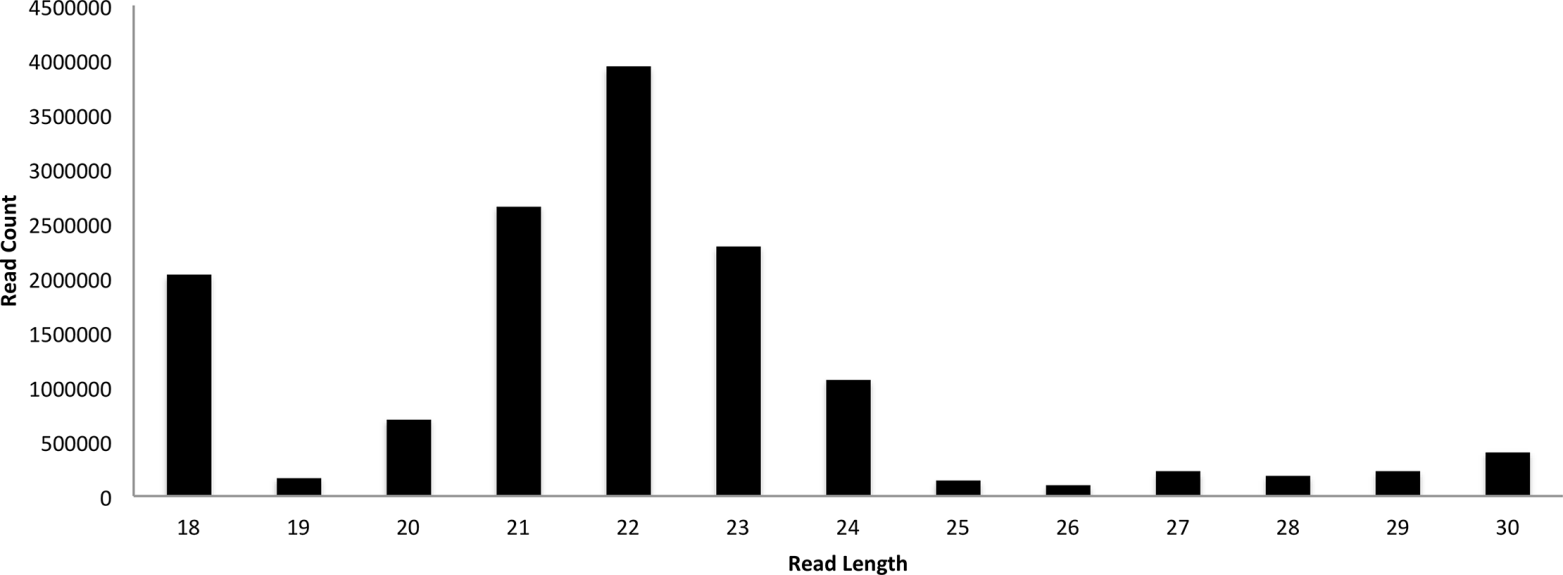
Length distribution of the total mappable reads.

To define the ovine miRNAome, we first generated a small-RNA library from sheep. Specifically, a small-RNA library was constructed from pooled RNA samples of nine tissues of adult male Dorset sheep (“global” sheep library; see Methods section for detailed approach). Moreover, to detect novel left ventricle specific miRNAs, a second small-RNA library (LV, “Left Ventricle specific” library) was generated from RNA samples obtained from the left ventricle of three adult male Dorset sheep. Illumina sequencing of the purified cDNA libraries generated 21,343,067 total raw reads: 9,512,054 from the LV library and 11,831,013 from the global library. Raw reads were processed by ACGT101-miR v4.2 (LC Sciences), a pipeline that employs a series of digital filters to remove unmappable reads [45,46]. Low-quality sequences, due to sample preparation, sequencing chemistry and processes, and the optical digital resolution of the sequencer detector, were removed. The remaining mappable sequences were grouped by unique families. A total of 5,537,088 and 9,089,864 mappable reads accounting for 58.2% and 76.8% of the total raw reads from LV and global libraries, respectively, were obtained. Fig 1 illustrates the length distribution of the total mappable reads, the majority of which were between 18 and 24 nt (90.93%).

**Fig 2:**
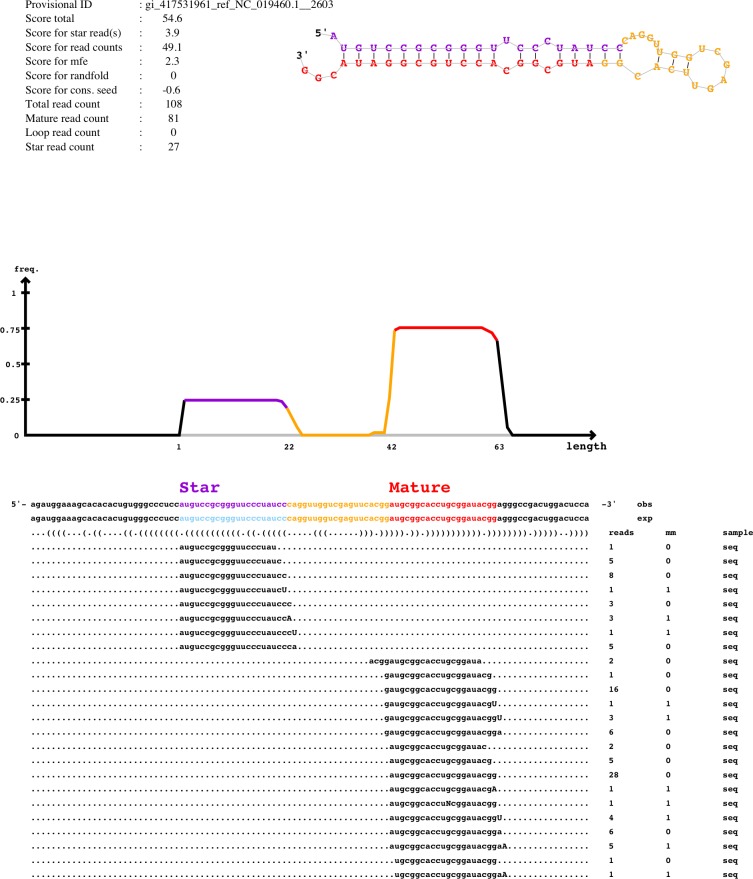
An example of the miRDeep2 output. The figure illustrates the output for the novel oar-miR-7134. The upper part shows the scores assigned to the miRNA, the reads count for the mature, star and loop sequences and the total count. The predicted secondary structure of the hairpin is also depicted, with the mature, star and loop sequences highlighted in different colors (red, purple and yellow, respectively). The bottom part of the figure shows all the reads associated to the miRNA, aligned to the mature, star and loop sequences of the predicted precursor on the genome (obs line) and the experimental sequence as reported in miRBase (exp line). For each sequence, the frequency (reads column) and mismatches with the genomic sequence (mm column) are given. The mismatches are also highlighted in capital letters. The different isomiRs, discussed in the Results and Discussion section, are extracted from this alignment data.

**Fig 3:**
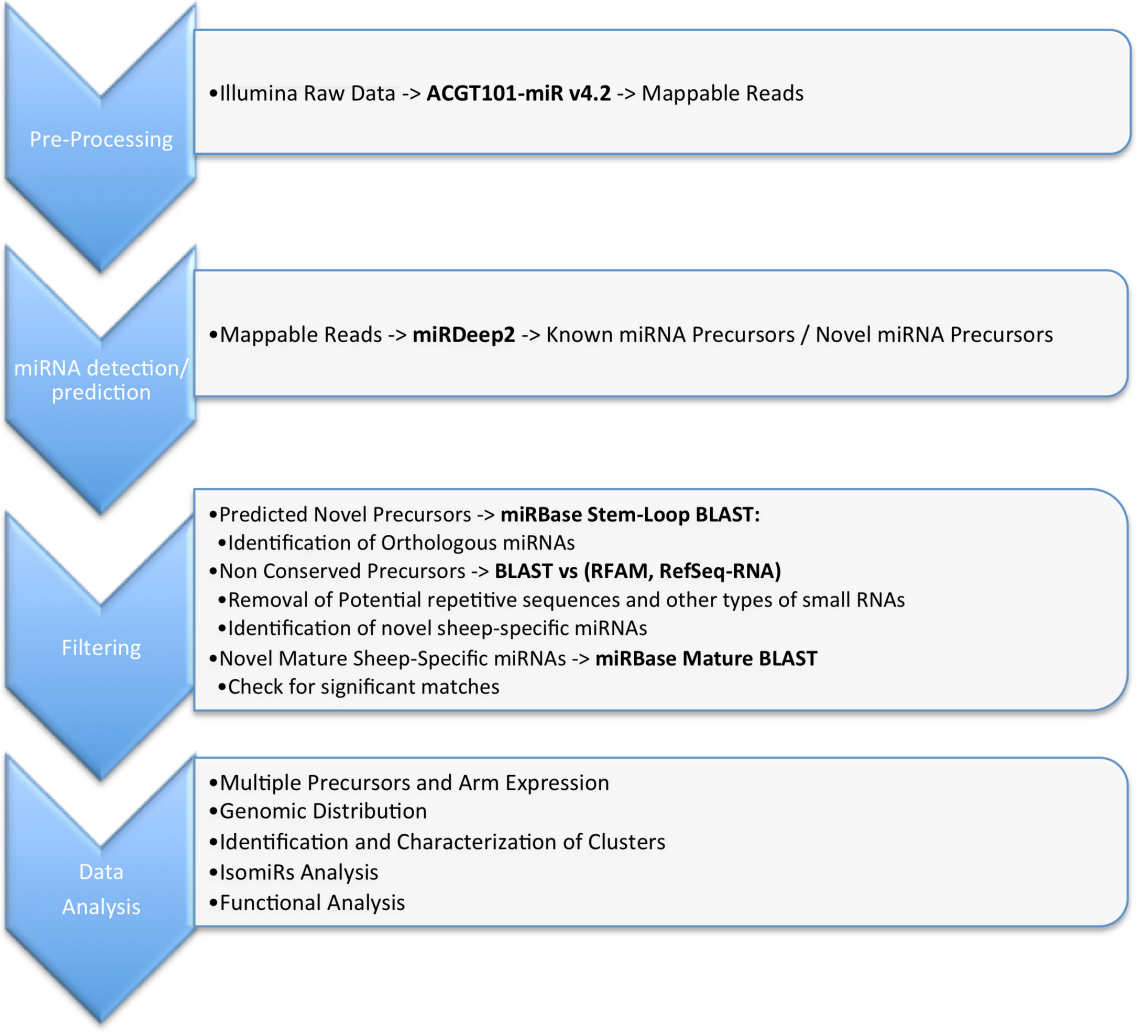
Computational pipeline of data analysis. The figure illustrates the four steps of the computational pipeline employed to analyze the RNAseq data. Pre-Processing: raw data were processed by the ACGT101-miR v4.2 pipeline in order to obtain good quality mappable reads. miRNA detection/prediction: this step was carried out by applying miRDeep2 to the mappable reads. The tool returned the lists of known and novel miRNA precursors identified. Filtering: the output of miRDeep2 was further analyzed by BLAST against different databases in order to assess evolutionary conservation of the predicted miRNAs and remove sequences matching other kinds of small RNAs. Data Analysis: last step consisted of the application of a series of ad-hoc scripts for the extraction of descriptive statistics. The tool IPA was used to perform the functional enrichment analysis of the targets of the identified miRNAs, which were predicted by the software miRiam.

Processed high-quality sequences were analyzed by miRDeep2 [47,48], a computational tool to map, analyze and score deep sequencing data for the identification of known and novel miRNAs. Fig 2 illustrates an example of the miRDeep2 output. miRDeep2 mapped a total of 3,927,458 reads from the LV library (70.93% of the total count) and 6,663,266 reads from the global library (73.30% of the total counts) to a mature 5p or 3p miRNA sequence. Fig 3 illustrates the computational pipeline employed for data analysis (results described below). The raw data has been submitted to the NCBI SRA database (Project accession #: SRP038892; Samples accession # LV: SRR1175693, OT: SRR1175694). The original output generated by miRDeep2 is available as supporting information (S1 File).

### Identification of Novel Sheep miRNAs

The miRDeep2 pipeline analysis identified 182 novel miRNA precursors, i.e. not previously reported in miRBase for sheep. A total of 1,607,604 reads from LV and 1,900,117 reads from the global library were mapped to novel miRNA candidates, accounting for 29% and 20.9% of the total mappable reads from LV and the global library, respectively.

### Novel Sheep miRNAs: Conservation Analysis

We analyzed putative precursor sequences by miRBase implementation of BLAST to assess their evolutionary conservation and identify potential orthologs. 110 precursors exhibited a significant similarity match with precursors from at least 5 other species (EValue < 4e-09), 9 showed a significant similarity with <5 species (EValue < 9e-05) and one was comparable to a distant precursor from *Drosophila pseudoobscura* (miR-219) (EValue = 2e-18). Moreover, 29 stem-loop sequences had a significant match with precursors from the large *Bos taurus* miRNA family miR-2284/2285 (EValue < 2e-04). This family contains 101 precursors recently detected by deep sequencing of *Bos taurus*, encoding highly similar mature products [36,49,50]. These are the first example of miR-2284/2285 homolog miRNAs in a species other than *Bos taurus*. The remaining 33 precursors were further analyzed by BLAST against the *Ovis aries* genome and the Refseq-RNA and Rfam databases [51]. This analysis removed 10 candidates. Four of ten were removed because they had more than five significant hits against different chromosomal areas of the sheep genome (E < 2e-08), hence likely to be repeated sequences. The other 6 showed significant similarity with other small RNAs such as small nuclear RNAs (snRNAs), small nucleolar RNAs (snoRNAs) and transfer RNAs (tRNAs). We classified the remaining 24 sequences as novel sheep-specific miRNA precursors.

The 172 conserved and non-conserved novel miRNA precursors encoded 262 unique novel 5p and 3p mature sequences (S1 Table). 84 precursors were detected in both LV and global samples, while 76 precursors were present in the global sample only, as opposed to 12 precursors which were instead specific to LV (Tables 1 and 2).

**Table 1:** Summary of novel sheep miRNAs.

Specificity	LV only	GL only	LV + GL	Total
**Precursors**	12	76	84	172
**Mature**	19	115	130	264

The table contains a detailed summary of the novel miRNAs discovered. It shows the number of precursors, and relative mature sequences, found in the Left Ventricle library (LV), the Global Library (GL) or both libraries (LV + GL).

**Table 2:** Conservation of novel sheep miRNAs.

Conservation	Conserved > = 5 species	Conserved < 5 species	Conserved 1 species	Non Conserved	Total
**Precursors**	112	10	29	21	172
**Mature**	177	16	45	26	264

The table shows the number of precursors, and relative mature sequences, which are non-conserved, conserved in more than five species, less than five species and in one species only. The latter include a distant homolog of miR-219 from *Drosophila pseudoobscura*, and the miRNAs of the miR-2284/2285 family from *Bos taurus*.

### Novel Sheep miRNAs: Arm Preference

Both arms of a miRNA precursor may give rise to functional levels of mature miRNA [15,52]. The dominant product may change from species to species and have different tissue expression preference, including normal versus pathological tissue [53–59]. Although 84 stem-loop sequences were detected in both samples, there were differences in the expression of their mature products. Notably, for 59 we detected the presence of the same mature sequences in both samples, as opposed to 25 precursors where the two samples differed in the presence of the corresponding mature products. For example, we detected both let-7e-5p and let-7e-3p in the global library, while only let-7e-5p was found in the LV library. Our data also showed that 86 out of 161 precursors found in global library (52.76%) and 51 out of 95 precursors found in LV (53.68%) expressed both 5p and 3p mature sequences at detectable levels (i.e. > 10 read counts). Moreover, 5p was the dominant product for more than half of the precursors in both LV (64.21%) and global library (53.98%), while only for five of 84 precursors a switch in arm preference between the two samples was observed.

A single mature miRNA can derive from multiple precursors [24]. This represents a common feature, which is widespread across evolution. Our analysis found 13 pairs of precursors, six exclusively present in the global library, each encoding the same mature sequence.

### Novel Sheep miRNAs: Nomenclature

Novel conserved miRNAs were named after their homologs in other species and assigned to their corresponding families, when possible (S1 Table). However, concerning the 29 precursors exhibiting significant similarity with the cow precursors belonging to the miR-2284/2285 family, the conservation analysis of their mature sequences returned multiple matches with members of the family. Therefore, such miRNAs were named after their closest matching mature sequences, taking specifically into account the seed area, when possible. In a few cases, we used the same name for highly similar sequences and added a progressive letter at the end (e.g. oar-miR-2285la and oar-miR-2285lb) (S1 Table).

We identified 24 novel sheep-specific miRNA precursors. Three were detected in both LV and global library, three were specific to LV and the remaining 18 were only detected in the global library. We performed a BLAST analysis of their mature sequences against the miRBase database, to check if they were related to any known miRNA from other species. Despite no significant match of its precursor with any other stem-loop sequence in miRBase, one mature sequence shared some similarity, including six bases in the seed region, with miR-359-3p from *Caenorhabditis briggsae*, thus we decided to use the same nomenclature. No significant homology to any miRNA from any other species was identified for the 23 remaining sequences, thus we assigned them provisional names oar-miR-N1 to oar-miR-N22. Two of these precursors encoded the same mature sequence, oar-miR-N14-3p, therefore they were named with the same number followed by the additional numbers 1 and 2 (oar-miR-N14-1, oar-miR-N14-2), according to miRBase nomenclature rules [60]. Most of the 23 precursors gave rise to one mature product only, while four had both arms expressed. However, most of the 26 mature sequences encoded by these precursors had a rather low reads count. In particular, 22 sequences had less than 100 reads, three had a read count between 100 and 1000, while only one of them, that was detected in the LV sample only, had > 1000 reads (oar-miR-4005-5p) (S1 Table).

### Identification of Known miRNAs in the Left Ventricle and Other Tissues

Our analysis also reported 100 known miRNA precursors from the global library encoding 159 mature sequences, 45 of which had not yet been reported in miRBase and were mostly non-dominant mature forms, i.e. the least expressed of the two mature products from a given precursor.

Specifically, 85 out of 100 precursors were also detected in LV, encoding a total of 126 mature sequences (S2 Table).

A total of 2,319,350 reads from LV and 4,762,811 reads from the global library were mapped to these miRNA mature sequences, accounting for 41.88% and 52.39% of the total mappable reads from LV and the global library, respectively.

The analysis was not able to account for 70 and 53 known mature miRNAs from LV and the global library, respectively. This could be due to the very rapid turnover rates of some miRNAs or to their specificity to different tissues than the ones considered in this study.

### Genomic Distribution

**Fig 4:**
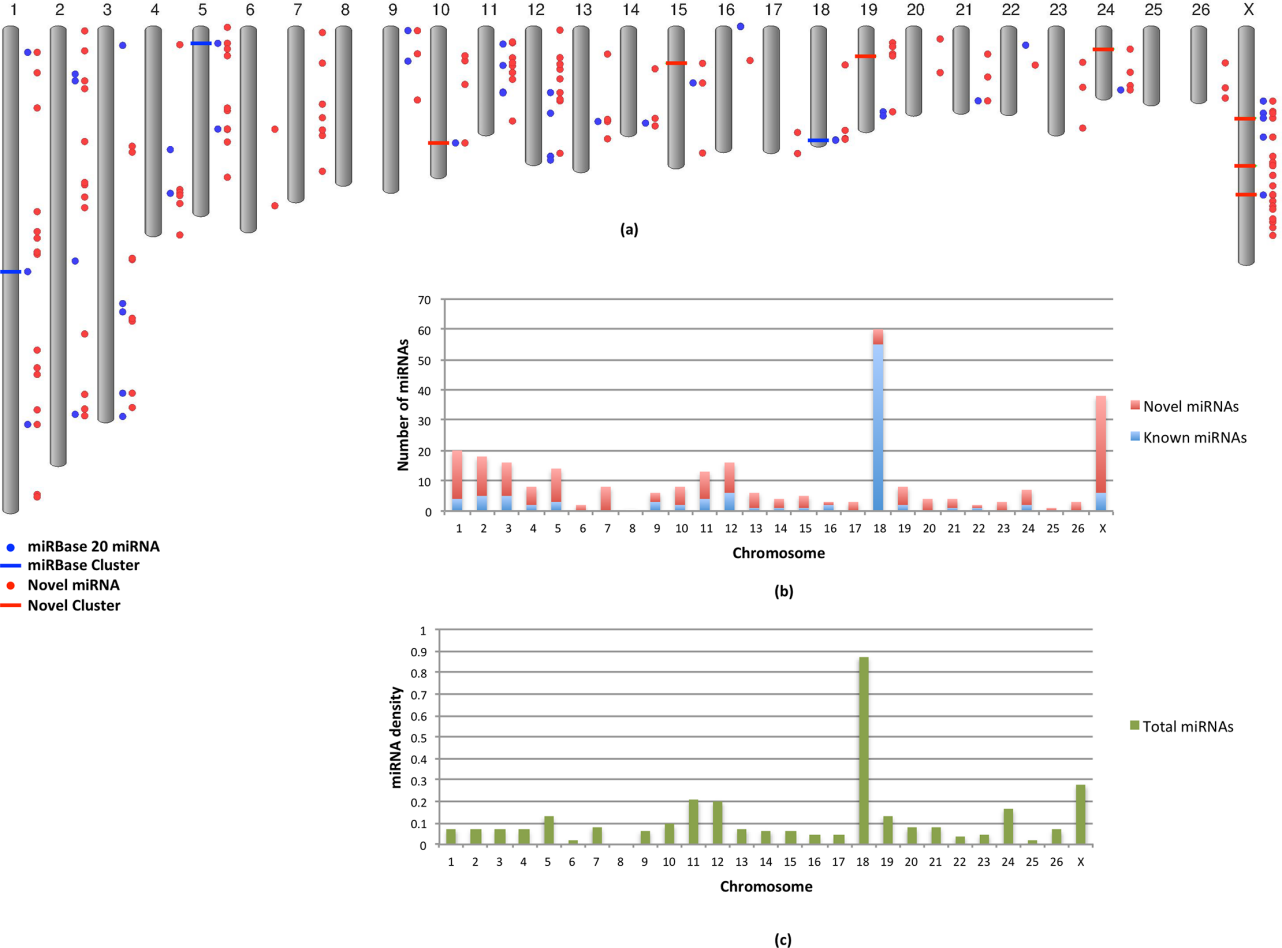
Genomic distribution of the identified miRNAs. (a) The figure shows the chromosomal distribution of the novel (red) and known (blue) miRNAs. The known and novel clusters are represented by blue and red bars, respectively. (b) The stacked column chart shows the total number of miRNAs per chromosome, highlighting the fractions of known (blue) and novel (red) ones. We report miRNAs on chromosomes 6, 7, 17, 20, 23, 25 and 26 for the first time. (c) The chart shows the miRNA density per chromosome, calculated as the number of miRNAs per Mbp (Megabase pair).

The novel miRNA precursors identified from our study were widely distributed throughout the genome with the exception of chromosome 8 where no miRNAs were located (Fig 4A). We discovered an average of 6.4 novel miRNAs/chromosome representing an ~3.5-fold increase in the number of miRNAs per chromosome (Fig 4B). The highest increase was of 6-fold on the X chromosome, where we identified 33 novel miRNAs, 26 of which were conserved in *Bos taurus* and other species on the X chromosome as well. Further, we discovered novel miRNAs on chromosomes 6, 7, 17, 20, 23, 25 and 26, where no miRNAs had been previously identified.

Overall, chromosome 18 had both the highest number of miRNAs (60) and highest density (0.87), calculated as the number of miRNAs per Mbp (Fig 4C). Five of 60 miRNAs on chromosome 18 were discovered in this study. Chromosome 6, and 22 had the lowest number of miRNAs (two each) and the lowest densities (0.017 and 0.039, respectively), if we exclude chromosome 8.

### Identification of Novel miRNA Clusters

A miRNA cluster is a group of precursors with an inter-miRNA distance of less than 10kb on the same genomic strand [24,61,62]. A miRNA cluster can be transcribed as a single polycistronic primary transcript and then processed into shorter pre-miRNAs to ultimately yield distinct mature miRNAs [63]. A simple analysis of the genomic locations of known sheep precursors recovered 10 clusters expressed in both samples and a large cluster spanning about 40 Kb on chromosome 18 containing 23 and 37 miRNA precursors expressed in LV and global library, respectively.

Moreover, the genomic analysis of novel miRNAs identified 7 novel miRNA clusters containing 17 precursors encoding a total of 30 mature miRNAs. With the exception of one cluster consisting of two precursors of miR-2285m, from the miR-2284 family, the novel clusters were evolutionarily conserved. In particular, the cluster miR-452/miR-3431 was found in the cow genome, while the remaining five clusters were conserved in several species, including cow and human. Three of the novel discovered miRNAs, miR-18a, miR-20a and miR-92a, belonged to the miR-17/92 cluster on chromosome 10, joining miR-17 and miR-19b that were already reported in miRBase. The miR-17/92 is a well-studied conserved cluster consisting of six miRNAs [64–66]. miR-19a was the only miRNA of the conserved cluster that we did not detect and that is not reported in miRBase to date.

Aside from the well-established oncogenic role played by the miRNAs of this cluster, miR-17-92 is implicated in both normal and pathological functions of the heart [66–68]. Specifically, the cluster regulates cardiomyocyte proliferation and dysregulation of this cluster during cardiovascular morphogenesis results in a lethal cardiomyopathy.

Three of the remaining conserved clusters that we identified were located on sheep chromosome X as well as their homologs in human and cow. Moreover, miR-222, which we found expressed in LV, joined miR-221 which was already reported in miRBase, to form a conserved cluster also on chromosome X.

Finally, we identified cluster mir-34b/c on chromosome 15 and mir-365a/193b on chromosome 24. The first was conserved in both human and cow, as well as in several other species, while the latter was conserved in human and gorilla only.

### IsomiRs

A common feature of miRNAs is the presence of different variants of the same mature sequence, called isomiRs. RNA-Seq has allowed more detailed characterization of this phenomenon, once thought due to experimental artifacts. Evidence shows that isomiRs are functional variants with a specific biological role [69,70]. Indeed, they have been frequently observed in many species, tissues and conditions and recent studies have reported cases of functional isomiRs exhibiting significant overlapping targeting properties with their reference counterpart [71]. Although many types of variations have been observed, isomiRs can be classified into three main categories: 5’ isomiRs, 3’ isomiRs and polymorphic isomiRs. The first two categories consist of shifted sequences which can also differ in length from the reference sequence and can be sub-classified into templated and non-templated, depending on whether the additional nucleotides at the 5’ or 3’ end do or do not match the reference stem-loop sequence, respectively. These variants appear to be the most commonly observed, especially the templated 3’-shifted variants, and are likely to derive from imprecise Drosha and Dicer cleavage [69]. Polymorphic isomiRs are rarer isoforms which exhibit internal nucleotide substitutions and are probably caused by editing events, such as A-to-I editing, which is the most prevalent type of RNA editing so far [72,73]. Given the crucial role of the seed sequence on miRNA function, the most interesting isomiR forms are those affecting the seed region, such as the 5’-shifted isomiRs and the seed-edited isomiRs.

**Fig 5:**
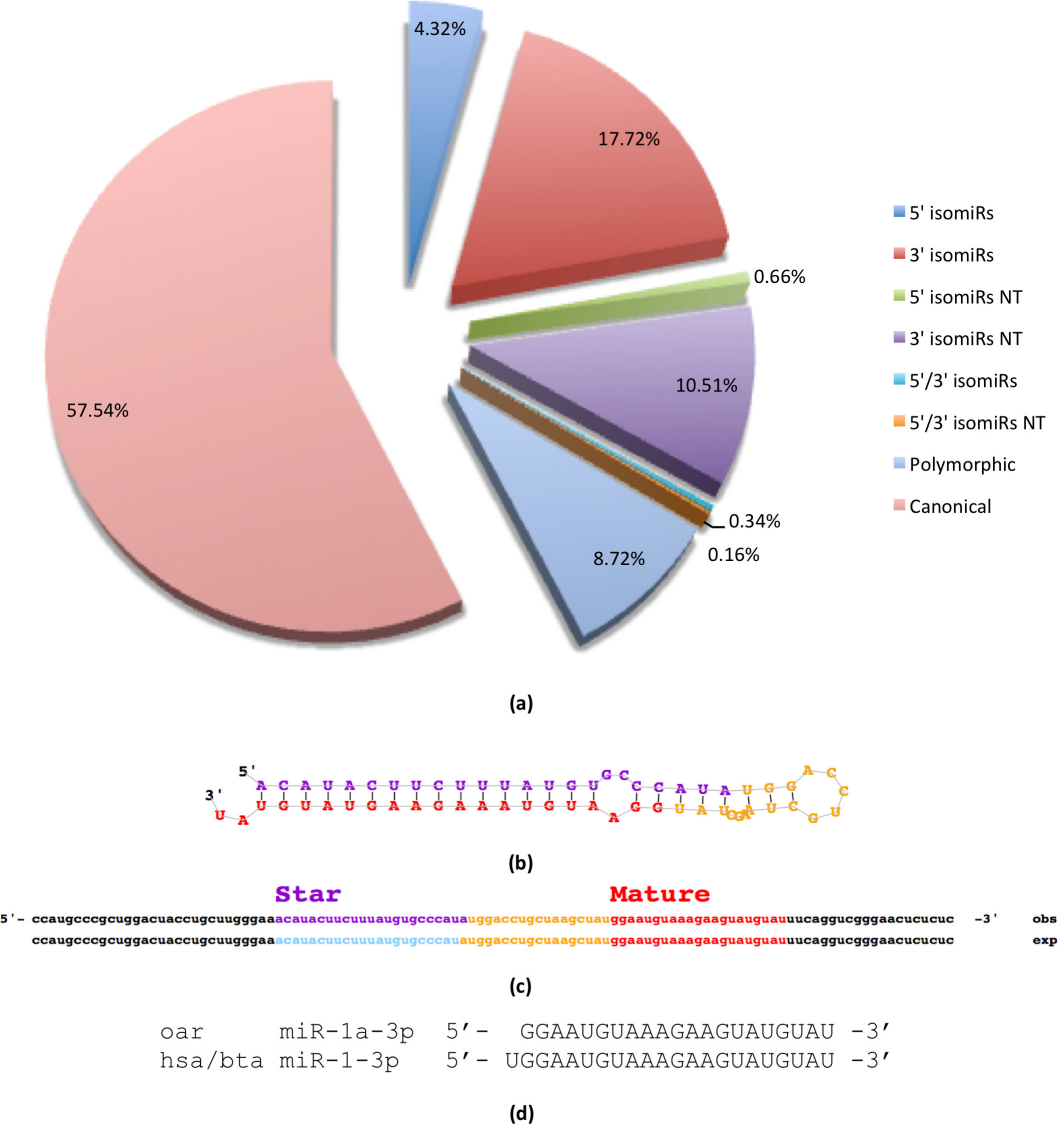
isomiRs distribution. (a) The figure shows the distribution of the different types of isomiRs calculated on the total of mapped reads from both samples (LV and global library). The reference form was predominant (57.54%), while the most frequent isomiR types were the templated and non-templated (NT) 3’ isomiR and the polymorphic isomiR. (b) The predicted precursor structure for oar-miR-1a. (c) The mature and star sequences of miR-1a identified by miRDeep2. (d) The dominant mature product of miR-1a is encoded in the 3p arm (oar-miR-1a-3p). It is a 5' isomiR of the conserved miR-1-3p, which lacks the first nucleotide (human and cow sequences are reported for comparison).

The analysis of our miRNA reads showed a predominance of the reference form of mature miRNAs in both the LV and the global samples (60.48% in LV, 55.70% in global library) although a significant percentage was present for other forms as well. The most frequent isomiR type identified in our data was the templated 3’ isomiR (extended/truncated/shifted) (13.54% in LV, 20.34% in global library), followed by the non-templated 3’ isomiR (9.65% in LV, 11.05% in global library), the polymorphic isomiR (9.39% in LV, 8.29% in global library) and the templated 5’ isomiR (extended/truncated/shifted) (5.67% in LV, 3.47% in global library). Non-templated 5’ isomiRs as well as templated and non-templated forms extended or truncated at both 5’ and 3’ ends were rare (< 1.3% in LV, < 1.2% in global library). Fig 5A illustrates the distribution of the different types of isomiRs in both samples.

For each novel identified conserved miRNA, the candidate mature products corresponded to either the miRBase reference versions or their 3’ isoforms, except for miR-1. Our analysis, indeed, detected 2 highly conserved precursors of miR-1 (EValue < 1e-18), for which the dominant mature product, as given rise by the 3p arm on both LV and global library, was a 5’-shifted isomiR of the conserved mature miR-1 (miR-1a-3p) (Fig 5B, 5C and 5D). In particular, this variant lacks the first nucleotide, thus affecting the seed area and, presumably, its targeting properties. We performed a functional analysis in order to investigate potential functional differences between our 5’-isomiR and its reference counterpart and the results are shown in the Analysis section.

### Validation of known and novel miRNAs by Stem-Loop qRT-PCR

In order to validate the expression of the novel candidate miRNAs described in the previous sections, we selected 10 miRNAs and performed Stem-Loop qRT-PCR on RNA from the LV sample. Stem-Loop qRT-PCR is a reliable and widely used method for the detection and quantification of mature miRNAs [74]. In particular, we selected 5 miRNAs that were detected in both the LV and OT samples (oar-miR-145-5p, oar-miR-146b-5p, oar-miR-378-3p, oar-miR-423-5p and miR-145-5p) and 5 miRNAs specifically observed in the LV sample only, including a non-conserved miRNA (oar-miR-222-3p, oar-miR-330-3p, oar-miR-324-5p, oar-miR-2285x-3p and oar-miR-4005-5p). The experiment confirmed the expression of all 10 miRNAs in LV, as shown in Table 3, thus validating the RNA-Seq and computational analysis. We also successfully validated the expression of 4 known miRNAs in the LV sample (oar-miR-21, oar-miR-493-5p, oar-miR-494-3p, oar-miR-133-3p) (see Table 3). More details on the experimental procedure and the probes used are given in the Methods section.

**Table 3:** Validation of known and novel miRNAs by Stem-Loop qRT-PCR.

**Novel miRNAs in LV**		
**miRNA**	**Ct**	**Std Dev**
miR-146b-5p	33.129	0.398256617
miR-151-5p	26.257	0.634608124
miR-378-3p	20.907	0.181923092
miR-423-5p	29.107	0.114140675
miR-145-5p	27.725	0.284937142
miR-222-3p	30.578	0.713898269
miR-330-3p	30.898	0.977237439
miR-324-5p	26.172	1.767929513
miR-2285x-3p	28.181	0.25621647
miR-4005-5p	28.181	0.25621647
*U6 snRNA (control)*	25.338	0.144719684
**Known miRNAs in LV**		
**miRNA**	**Ct**	**Std Dev**
miR-21-5p	26.25	0.221885857
miR-493-5p	34.25	0.41585374
miR-493-4p	21.73	1.291861135
miR-133-3p	14.44	2.278697632
*U6 snRNA (control)*	18.59	1.383107503

The experiments confirmed the expression of 10 novel and 4 known miRNAs in the left ventricle (LV). For each miRNA and the control U6, the table reports the threshold cycle (Ct) and the standard deviation (Std Dev).

### Analysis of Left-Ventricle Specific miRNAs

Our data allowed the discovery and characterization of novel miRNAs in the ovine left-ventricle and in other tissues. We primarily focused this study on microRNAs of sheep left ventricle, as this is the integral cardiac chamber involved in the development of heart failure. The ovine model has been an appropriate surrogate to study human heart failure, as it closely resembles the human heart in size, coronary anatomy as well as a predictable pattern of left ventricular remodeling following a myocardial infarction. As part of a larger project designed to look at both control tissue from a normal sheep heart and that of an infarcted, diseased heart, we sought to first create a roadmap of the normal miRNAome of the sheep–particularly the left ventricle.

Further, our results recovered miRNAs that were already deposited in miRBase. Additional quantitative analysis will be necessary to assess significant differential expression of the detected miRNAs in the tissues examined [75]. However, large differences in the reads count of a miRNA between two different conditions may provide a meaningful estimate of its tissue-specificity [40].

We performed a simple fold-change analysis with the tool DESeq, that allows the analysis of RNA-Seq data from experiments without replicates [76].

Among the known miRNAs, miR-133a-3p, miR-22-3p and miR-26b, showed a much higher expression in LV compared to global library, in terms of normalized reads count (Fold Changes were 7.87, 4.29, and 4.07, respectively). These miRNAs were already known to be crucially involved in cardiac functions and diseases in human. In particular, miR-133 is typically enriched in cardiac and skeletal muscle and is involved in cell specification, differentiation and development [77]. Moreover, miR-133 has been proven to restrict injury-induced cardiomyocyte proliferation and its down-regulation is a prerequisite for the initiation of apoptosis, the development of fibrosis, and prolongation of the QT interval [77,78]. miR-22 and miR-26b had the second and third highest fold-changes. Recent studies showed that miR-22 functions as an integrator of Ca(2+) homeostasis and myofibrillar protein content during stress in the heart and that it has a role as a critical regulator of cardiomyocyte hypertrophy and cardiac remodeling [79,80]. miR-26b is known to be involved in cardiac hypertrophy as well, its down-regulation being required for the induction of pressure-induced cardiac hypertrophy [81].

A functional enrichment analysis of the predicted targets for miR-133a-3p, miR-22-3p and miR-26b performed by using the tool IPA (Ingenuity^®^ Systems, www.ingenuity.com), indeed reflected their proven role in heart specific functions and diseases, being *Cardiovascular Disease* reported among the top 5 Diseases and Disorders (P < 0.01). In particular, the significantly associated terms included *Myocardial Infarction* (P < 0.0001), *Ischemia of Heart* (P < 0.001) and *Proliferation of Cardiac Fibroblasts* (P < 0.05). *Cardiac Hypertrophy Signaling* was reported among the pathways in which the three miRNAs could be significantly involved (P < 0.05), while the associated IPA toxicity list included terms such as *Increases Cardiac Proliferation* (P< 0.0001), *Increases Cardiac Dilation* (P< 0.01) and *Cardiac Necrosis/Cell Death* (P< 0.05). The most significant predicted targets involved in heart specific functions and diseases included IL6, ITGB2, PTGS1, PTGS2, ADRB3 and MAPK13 (see S3 Table).

Our analysis also showed higher expression of miR-125b in LV compared to the global library. miR-125b is a known miRNA in sheep. A recent study demonstrated that the overexpression of this miRNA during human embryonic stem cell (hESC) differentiation into myocardial precursors and cardiomyocites (CM) results in the up-regulation of early cardiac transcription factors and accelerates progression of hESC-derived myocardial precursors to an embryonic CM phenotype [82]. Moreover, miR-125b was reported to protect the myocardium from ischaemia/reperfusion injury in mouse by preventing p53-mediated apoptotic signaling and suppressing TRAF6-mediated NF-kB activation [83].

Among the novel ovine miRNAs detected in our study, miR-208b showed higher expression in LV compared to the global library. miR-208b is a well known “myomiR” in human, that is, a muscle-specific miRNA, normally found highly expressed in cardiac tissue. This miRNA plays an important role in muscle growth due to its involvement in myogenesis and slow myosin heavy chain gene regulation [84,85].

The results of our analysis are also consistent with a recent work introducing the heart structure-specific transcriptomic atlas from rat, dog and monkey, where the authors identified, among others, miR-133a, miR-208b and miR-1 (which is discussed later in this section) as highly expressed in myocardial tissue, including the LV [86].

None of the 11 LV-specific miRNAs identified in our study was reported as heart-specific in human before. However, for two of them, miR-222 and miR-216b, a correlation with heart functions and disease was previously reported. One study found that miR-222 is necessary for exercise-induced cardiac growth and protects against pathological cardiac remodeling [87]. Another recent study reported that miR-221/222 contribute to the atherogenic calcification of vascular smooth muscle cells [88]. miR-216b was reported to be up-regulated in rat heart following treatment with Doxorubicin and preceding the rise of overt lesions, and may represent a valuable early genomic biomarker of drug-induced cardiac injury [89].

We performed a functional enrichment analysis of the predicted targets for the 11 LV-specific novel conserved and non-conserved miRNAs identified in our study, by using IPA. The results included *Cardiovascular System Development and Function* among the top 5 Physiological System Development and Function classes. In particular, the analysis identified 13 different function terms, including *Vasculogenesis* (P < 0.001), *Activation And Permeability Of Microvascular Endothelial Cells* (P < 0.01) and *Chemotaxis Of Vascular Endothelial Cells* (P < 0.001). Other significant terms associated to the LV-specific miRNAs included *Heart Disease* (P < 0.001), *Apoptosis* (P < 0.0001) and *Apoptosis Of Vascular Endothelial Cells* (P < 0.01).

The associated IPA toxicity list included heart-specific terms such as *Cardiac Hypertrophy* (P < 0.05), *Cardiac Hypertrophy Signaling* (P < 0.05), *Cardiac Fibrosis* (P < 0.0001), *Cardiac Necrosis/Cell Death* (P < 0.05) and *Increases Cardiac Dilation* (P < 0.0001), while the significant tox functions included *Myocardial Infarction* (P < 0.01). The most significant predicted targets enriched in cardiovascular system functions and tox terms were ID3, PLAU, TIMP1, IL8, IL2RA, PTGS2, IFNG and SOD2 (S3 Table).

Finally, we performed a functional analysis of miR-1-3p and its 5’ isomiR, oar-miR-1a-3p, the latter detected as the predominant mature form of miR-1 in both the LV and global samples. miR-1 is well known to play a key role in the development and physiology of muscle tissues including the heart. Interestingly, a recent paper reported a miR-1 polymorphic isomiR, different than the one identified in our study, significantly expressed in the heart left ventricle of the rat [90]. Therefore, we wanted to make some functional hypothesis on the potential biological roles of this newly discovered isomiR. The analysis reported both overlapping and specific targets for the two forms. In particular, genes such as AGTR1, ANKRD1, CAV2, IL13 and KDR, all predicted targets for the 5’ isomiR, were associated to the *Cardiovascular System Development and Function* network, which was reported among the 5 top scoring networks specific for the 5’-isomiR. Both mature forms shared Disease and Function terms such as *Cardiovascular Disease* (P < 0.0028), *Organismal Injury and Abnormalities* (P < 0.0028), *Hematological System Development and Function* (P < 0.0028) and *Cellular Growth and Proliferation* (P < 0.0028), while the isomiR was also specifically enriched in terms such as *Cancer* and *Skeletal and Muscular Disorders* (P < 0.003), and the *IL-8 signaling pathway* (P < 0.002) (S3 Table). Our analysis suggests potential heart-specific functions of this isomiR and encourage further experimental investigation, which we will undertake in the next phase of our project.

## Conclusions

Our results define the miRNA signature of sheep left ventricle. Our findings validate previously discovered miRNAs, as well as identify novel miRNAs in heart. Further, our data provide new information on the identity of LV-specific miRNAs in sheep LV. We postulate that these findings will be crucial for our understanding of the molecular mechanisms of protein and cell regulation in this critical pre-clinical model.

## Supporting Information

S1 File miRDeep2 file. The non-processed original miRDeep2 output.
(ZIP)

S1 Table Novel miRNAs. Details include reads count in the two samples, genomic coordinates, conservation, sequences and clusters. For each miRNA, the original miRDeep2 ID, matching the corresponding entry in S1 File, is provided.
(XLSX)

S2 Table Known miRNAs. Details include reads count in the two samples, genomic coordinates and sequences. For each miRNA, the original miRDeep2 ID, matching the corresponding file name, is provided.
(XLSX)

S3 Table Functional Analysis computed by IPA. (a) LV biological functions, (b) LV tox functions, (c) LV pathways, (d) LV networks, (e) LV toxicity lists, (f) miR-133a-3p, miR-22-3p and miR-26b biological functions, (g) miR-133a-3p, miR-22-3p and miR-26b tox functions, (h) miR-133a-3p, miR-22-3p and miR-26b pathways, (i) miR-133a-3p, miR-22-3p and miR-26b networks, (j) miR-133a-3p, miR-22-3p and miR-26b toxicity lists, (k) reference miR-1 biological functions, (l) reference miR-1 pathways, (m) reference miR-1 networks, (n) miR-1a biological functions, (o) miR-1a pathways, (p) miR-1a networks.
(XLSX)

## References

[1] Roger VL, Go AS, Lloyd-Jones DM, Adams RJ, Berry JD, Brown TM, et al Heart Disease and Stroke Statistics—2011 Update: A Report From the American Heart Association. Circulation. 2011;123: e18–e209. 10.1161/CIR.0b013e3182009701 21160056 PMC4418670

[2] Fang J, Mensah GA, Croft JB, Keenan NL. Heart Failure-Related Hospitalization in the U.S., 1979 to 2004. Journal of the American College of Cardiology. 2008;52: 428–434. 10.1016/j.jacc.2008.03.061 18672162

[3] Oz MC. Surgical issues in heart failure: What's new? J Card Fail. 2001;7: 18–24. 10.1054/jcaf.2001.26654 11605162

[4] Mahon NG, O'Neill JO, Young JB, Bennett R, Hoercher K, Banbury MK, et al Contemporary outcomes of outpatients referred for cardiac transplantation evaluation to a tertiary heart failure center: impact of surgical alternatives. J Card Fail. 2004;10: 273–278. 10.1016/j.cardfail.2003.11.001 15309691

[5] Spata T, Bobek D, Whitson BA, Parthasarathy S, Mohler PJ, Higgins RSD, et al A nonthoracotomy myocardial infarction model in an ovine using autologous platelets. BioMed research international. 2013;2013: 938047–938047. 10.1155/2013/938047 24367790 PMC3866830

[6] Monnet E, Chachques JC. Animal Models of Heart Failure: What Is New? The Annals of Thoracic Surgery. 2005;79: 1445–1453. 10.1016/j.athoracsur.2004.04.002 15797108

[7] Kim W, Jeong MH, Sim DS, Hong YJ, Song HC, Park JT, et al A porcine model of ischemic heart failure produced by intracoronary injection of ethyl alcohol. Heart Vessels. 2010;26: 342–348. 10.1007/s00380-010-0022-3 20963597

[8] Krombach GA, Kinzel S, Mahnken AH, Günther RW, Buecker A. Minimally Invasive Close-Chest Method for Creating Reperfused or Occlusive Myocardial Infarction in Swine. Invest Radiol. 2005;40: 1–5. 15597015

[9] Kim WG, Shin YC, Hwang CL, Na CY. Comparison of myocardial infarction with sequential ligation of the left anterior descending artery and its diagonal branch in dogs and sheep. the international journal of artificial organs. 2003;26: 1–7. 10.1177/039139880302600411 12757035

[10] Dixon JA, Spinale FG. Large Animal Models of Heart Failure: A Critical Link in the Translation of Basic Science to Clinical Practice. Circ Heart Fail. 2009;2: 262–271. 10.1161/CIRCHEARTFAILURE.108.814459 19808348 PMC2762217

[11] Locatelli P, Olea FD, Mendiz O, Salmo F, Fazzi L, Hnatiuk A, et al An ovine model of postinfarction dilated cardiomyopathy in animals with highly variable coronary anatomy. ILAR J. 2011;52: E16–21. 21454923 10.1093/ilar.52.1.e16

[12] Moainie SL, Gorman JH, Guy TS, Bowen FW, Jackson BM, Plappert T, et al An Ovine Model of Postinfarction Dilated Cardiomyopathy. Ann Thorac Surg. 2002;74: 753–760. 12238835 10.1016/s0003-4975(02)03827-4

[13] Weaver ME, Pantely GA, Bristow JD, Ladley HD. A quantitative study of the anatomy and distribution of coronary arteries in swine in comparison with other animals and man. Cardiovasc Res. 1986;20: 907–917. 3802126 10.1093/cvr/20.12.907

[14] Bartel DP. MicroRNAs: genomics, biogenesis, mechanism, and function. Cell. 2004;116: 281–297. 14744438 10.1016/s0092-8674(04)00045-5

[15] Du T. microPrimer: the biogenesis and function of microRNA. Development. 2005;132: 4645–4652. 10.1242/dev.02070 16224044

[16] Bartel DP. MicroRNAs: Target Recognition and Regulatory Functions. Cell. 2009;136: 215–233. 10.1016/j.cell.2009.01.002 19167326 PMC3794896

[17] Tili E, Michaille J-J, Croce CM. MicroRNAs play a central role in molecular dysfunctions linking inflammation with cancer. Immunol Rev. 2013;253: 167–184. 10.1111/imr.12050 23550646

[18] Croce CM. Causes and consequences of microRNA dysregulation in cancer. Nat Rev Genet. 2009;10: 704–714. 10.1038/nrg2634 19763153 PMC3467096

[19] Soifer HS, Rossi JJ, Saetrom P. MicroRNAs in Disease and Potential Therapeutic Applications. Mol Ther. 2007;15: 2070–2079. 10.1038/sj.mt.6300311 17878899

[20] Sayed D, Abdellatif M. MicroRNAs in Development and Disease. Physiol Rev. 2011;91: 827–887. 10.1152/physrev.00006.2010 21742789

[21] Sen Matsumoto, Sakata Y, Suna S, Nakatani D, Usami M, Hara M, et al Circulating p53-Responsive MicroRNAs Are Predictive Indicators of Heart Failure After Acute Myocardial Infarction. Circulation Research. 2013;113: 322–326. 10.1161/CIRCRESAHA.113.301209/-/DC1 23743335

[22] Li C, Fang Z, Jiang T, Zhang Q, Liu C, Zhang C, et al Serum microRNAs profile from genome-wide serves as a fingerprint for diagnosis of acute myocardial infarction and angina pectoris. BMC Med Genomics. 2013;6. 10.1186/1755-8794-6-16 PMC3655858 23641832

[23] Divakaran V, Mann DL. The Emerging Role of MicroRNAs in Cardiac Remodeling and Heart Failure. Circulation Research. 2008;103: 1072–1083. 10.1161/CIRCRESAHA.108.183087 18988904 PMC3982911

[24] Kozomara A, Griffiths-Jones S. miRBase: integrating microRNA annotation and deep-sequencing data. Nucleic Acids Res. 2011;39: D152–D157. 10.1093/nar/gkq1027 21037258 PMC3013655

[25] Kozomara A, Griffiths-Jones S. miRBase: integrating microRNA annotation and deep-sequencing data. Nucleic Acids Res. 2010;39: D152–D157. 10.1093/nar/gkq1027 21037258 PMC3013655

[26] Sheng X, Song X, Yu Y, Niu L, Li S, Li H, et al Characterization of microRNAs from sheep (Ovis aries) using computational and experimental analyses. Mol Biol Rep. 2010;38: 3161–3171. 10.1007/s11033-010-9987-3 20140706

[27] Galio L, Droineau S, Yeboah P, Boudiaf H, Bouet S, Truchet S, et al MicroRNA in the ovine mammary gland during early pregnancy: spatial and temporal expression of miR-21, miR-205, and miR-200. Physiological Genomics. 2013;45: 151–161. 10.1152/physiolgenomics.00091.2012 23269700

[28] Caiment F, Charlier C, Hadfield T, Cockett N, Georges M, Baurain D. Assessing the effect of the CLPG mutation on the microRNA catalog of skeletal muscle using high-throughput sequencing. Genome Res. 2010;20: 1651–1662. 10.1101/gr.108787.110 20944086 PMC2989991

[29] Zhang S, Zhao F, Wei C, Sheng X, Ren H, Xu L, et al Identification and Characterization of the miRNA Transcriptome of Ovis aries. PLoS ONE. Public Library of Science; 2013;8: e58905 10.1371/journal.pone.0058905 23516575 PMC3596360

[30] Barozai MYK. The novel 172 sheep (Ovis aries) microRNAs and their targets. Mol Biol Rep. Springer Netherlands; 2012;39: 6259–6266. 10.1007/s11033-012-1446-x 22302387

[31] Krombach GA, Kinzel S, Mahnken AH, Günther RW, Buecker A. Minimally invasive close-chest method for creating reperfused or occlusive myocardial infarction in swine. Invest Radiol. 2005;40: 14–18. 15597015

[32] Li M, Xia Y, Gu Y, Zhang K, Lang Q, Chen L, et al MicroRNAome of porcine pre- and postnatal development. Sorensen TIA, editor. PLoS ONE. Public Library of Science; 2010;5: e11541 10.1371/journal.pone.0011541 20634961 PMC2902522

[33] Wei Z, Liu X, Feng T, Chang Y. Novel and conserved micrornas in Dalian purple urchin (Strongylocentrotus nudus) identified by next generation sequencing. Int J Biol Sci. 2011;7: 180–192. 21383954 10.7150/ijbs.7.180 PMC3048847

[34] Friedlander MR, Mackowiak SD, Li N, Chen W, Rajewsky N. miRDeep2 accurately identifies known and hundreds of novel microRNA genes in seven animal clades. Nucleic Acids Res. 2011;40: 37–52. 10.1093/nar/gkr688 21911355 PMC3245920

[35] Langmead B, Trapnell C, Pop M, Salzberg SL. Ultrafast and memory-efficient alignment of short DNA sequences to the human genome. Genome Biol. 2009;10: R25 10.1186/gb-2009-10-3-r25 19261174 PMC2690996

[36] Huang J, Ju Z, Li Q, Hou Q, Wang C, Li J, et al Solexa sequencing of novel and differentially expressed microRNAs in testicular and ovarian tissues in Holstein cattle. Int J Biol Sci. 2011;7: 1016–1026. 21912509 10.7150/ijbs.7.1016 PMC3164151

[37] Friedländer MR, Chen W, Adamidi C, Maaskola J, Einspanier R, Knespel S, et al Discovering microRNAs from deep sequencing data using miRDeep. Nat Biotechnol. 2008;26: 407–415. 10.1038/nbt1394 18392026

[38] Liao J-Y, Ma L-M, Guo Y-H, Zhang Y-C, Zhou H, Shao P, et al Deep Sequencing of Human Nuclear and Cytoplasmic Small RNAs Reveals an Unexpectedly Complex Subcellular Distribution of miRNAs and tRNA 3′ Trailers. Xu S-Y, editor. PLoS ONE. 2010;5: e10563 10.1371/journal.pone.0010563 20498841 PMC2871053

[39] Sharbati S, Friedländer MR, Sharbati J, Hoeke L, Chen W, Keller A, et al Deciphering the porcine intestinal microRNA transcriptome. BMC Genomics. 2010;11: 275 10.1186/1471-2164-11-275 20433717 PMC2873480

[40] Andreassen R, Worren M, Hoyheim B. Discovery and characterization of miRNA genes in atlantic salmon (Salmo salar) by use of a deep sequencing approach. BMC Genomics. 2013;14: 482 10.1186/1471-2164-14-482 23865519 PMC3728263

[41] Metpally RPR, Nasser S, Malenica I, Courtright A, Carlson E, Ghaffari L, et al Comparison of Analysis Tools for miRNA High Throughput Sequencing Using Nerve Crush as a Model. Front Gene. 2013;4 10.3389/fgene.2013.00020 PMC3585423 23459507

[42] Dhahbi JM, Atamna H, Boffelli D, Magis W, Spindler SR, Martin DIK. Deep Sequencing Reveals Novel MicroRNAs and Regulation of MicroRNA Expression during Cell Senescence. Hansen IA, editor. PLoS ONE. 2011;6: e20509–12. 10.1371/journal.pone.0020509 21637828 PMC3102725

[43] Hu HY, Guo S, Xi J, Yan Z, Fu N, Zhang X, et al MicroRNA Expression and Regulation in Human, Chimpanzee, and Macaque Brains. Stubbs L, editor. PLoS Genet. 2011;7: e1002327 10.1371/journal.pgen.1002327 22022286 PMC3192836

[44] Lagana A, Forte S, Russo F, Giugno R, Pulvirenti A, Ferro A. Prediction of human targets for viral-encoded microRNAs by thermodynamics and empirical constraints. J RNAi Gene Silencing. 2010;6: 379 20628498 PMC2902144

[45] Li M, Xia Y, Gu Y, Zhang K, Lang Q, Chen L, et al MicroRNAome of porcine pre-and postnatal development. PLoS ONE. 2010. 10.1371/journal.pone.0011541 PMC2902522 20634961

[46] Wei Z, Liu X, Feng T, Chang Y. Novel and conserved micrornas in Dalian purple urchin (Strongylocentrotus nudus) identified by next generation sequencing. Int J Biol Sci. 2011. 10.7150/ijbs.7.180 PMC3048847 21383954

[47] Friedländer MR, Mackowiak SD, Li N, Chen W, Rajewsky N. Nucleic Acids Res. 2012;40. 10.1093/nar/gkr688 PMC3245920 21911355

[48] Friedländer MR, Chen W, Adamidi C, Maaskola J, Einspanier R, Knespel S, et al Nat Biotechnol. 2008. 10.1038/nbt1394 18392026

[49] Glazov EA, Kongsuwan K, Assavalapsakul W, Horwood PF, Mitter N, Mahony TJ. Repertoire of Bovine miRNA and miRNA-Like Small Regulatory RNAs Expressed upon Viral Infection. Randau L, editor. PLoS ONE. 2009;4: e6349–6. 10.1371/journal.pone.0006349 19633723 PMC2713767

[50] Muroya S, Taniguchi M, Shibata M, Oe M. Profiling of differentially expressed microRNA and the bioinformatic target gene analyses in bovine fast-and slow-type muscles by massively parallel sequencing. Journal of animal …. 2013. 10.2527/jas.2012-5371 23100578

[51] Burge SW, Daub J, Eberhardt R, Tate J, Barquist L, Nawrocki EP, et al Rfam 11.0: 10 years of RNA families. Nucleic Acids Res. 2012;41: D226–D232. 10.1093/nar/gks1005 23125362 PMC3531072

[52] Rana TM. Illuminating the silence: understanding the structure and function of small RNAs. Nat Rev Mol Cell Biol. 2007;8: 23–36. 10.1038/nrm2085 17183358

[53] Griffiths-Jones S, Hui JHL, Marco A, Ronshaugen M. MicroRNA evolution by arm switching. EMBO Rep. 2011;12: 172–177. 10.1038/embor.2010.191 21212805 PMC3049427

[54] Ro S, Park C, Young D, Sanders KM, Yan W. Tissue-dependent paired expression of miRNAs. Nucleic Acids Res. 2007;35: 5944–5953. 10.1093/nar/gkm641 17726050 PMC2034466

[55] Ruby JG, Stark A, Johnston WK, Kellis M, Bartel DP, Lai EC. Evolution, biogenesis, expression, and target predictions of a substantially expanded set of Drosophila microRNAs. Genome Res. 2007;17: 1850–1864. 10.1101/gr.6597907 17989254 PMC2099593

[56] de Wit E, Linsen SEV, Cuppen E, Berezikov E. Repertoire and evolution of miRNA genes in four divergent nematode species. Genome Res. 2009;19: 2064 10.1101/gr.093781.109 19755563 PMC2775598

[57] Chiang HR, Schoenfeld LW, Ruby JG, Auyeung VC, Spies N, Baek D, et al Mammalian microRNAs: experimental evaluation of novel and previously annotated genes. Genes Dev. 2010;24: 992–1009. 10.1101/gad.1884710 20413612 PMC2867214

[58] Marco A, Macpherson JI, Ronshaugen M, Griffiths-Jones S. MicroRNAs from the same precursor have different targeting properties. Silence. Silence; 2012;3: 1–1. 23016695 10.1186/1758-907X-3-8 PMC3503882

[59] Li S-C, Tsai K-W, Pan H-W, Jeng Y-M, Ho M-R, Li W-H. MicroRNA 3' end nucleotide modification patterns and arm selection preference in liver tissues. BMC Syst Biol. 2012;6: S14 10.1186/1752-0509-6-S2-S14 PMC3521178 23282006

[60] Ambros V, Bartel B, Bartel DP, Burge CB, Carrington JC, Chen X, et al A uniform system for microRNA annotation. RNA. 2003;9: 277–279. 12592000 10.1261/rna.2183803 PMC1370393

[61] Altuvia Y. Clustering and conservation patterns of human microRNAs. Nucleic Acids Res. 2005;33: 2697–2706. 10.1093/nar/gki567 15891114 PMC1110742

[62] Tanzer A, Stadler PF. Molecular Evolution of a MicroRNA Cluster. J Mol Biol. 2004;339: 327–335. 10.1016/j.jmb.2004.03.065 15136036

[63] Saini HK, Griffiths-Jones S, Enright AJ. Genomic analysis of human microRNA transcripts. Proceedings of the National Academy of Sciences. National Acad Sciences; 2007;104: 17719–17724. 10.1073/pnas.0703890104 PMC2077053 17965236

[64] Hayashita Y. A Polycistronic MicroRNA Cluster, miR-17-92, Is Overexpressed in Human Lung Cancers and Enhances Cell Proliferation. Cancer Res. 2005;65: 9628–9632. 10.1158/0008-5472.CAN-05-2352 16266980

[65] Ventura A, Young AG, Winslow MM, Lintault L, Meissner A, Erkeland SJ, et al Targeted Deletion Reveals Essential and Overlapping Functions of the miR-17∼92 Family of miRNA Clusters. Cell. 2008;132: 875–886. 10.1016/j.cell.2008.02.019 18329372 PMC2323338

[66] Mendell JT. miRiad Roles for the miR-17-92 Cluster in Development and Disease. Cell. 2008;133: 217–222. 10.1016/j.cell.2008.04.001 18423194 PMC2732113

[67] Chen J, Huang Z-P, Seok HY, Ding J, Kataoka M, Zhang Z, et al mir-17-92 cluster is required for and sufficient to induce cardiomyocyte proliferation in postnatal and adult hearts. Circulation Research. 2013;112: 1557–1566. 10.1161/CIRCRESAHA.112.300658 23575307 PMC3756475

[68] Danielson LS, Park DS, Rotllan N, Chamorro-Jorganes A, Guijarro MV, Fernandez-Hernando C, et al Cardiovascular dysregulation of miR-17-92 causes a lethal hypertrophic cardiomyopathy and arrhythmogenesis. The FASEB Journal. 2013;27: 1460–1467. 10.1096/fj.12-221994 23271053 PMC3606524

[69] Neilsen CT, Goodall GJ, Bracken CP. IsomiRs—the overlooked repertoire in the dynamic microRNAome. Trends Genet. 2012;28: 544–549. 10.1016/j.tig.2012.07.005 22883467

[70] Cloonan N, Wani S, Xu Q, Gu J, Lea K, Heater S, et al MicroRNAs and their isomiRs function cooperatively to target common biological pathways. Genome Biol. BioMed Central Ltd; 2011;12: R126 10.1186/gb-2011-12-12-r126 PMC3334621 22208850

[71] Llorens F, Bañez-Coronel M, Pantano L, Del Río JA, Ferrer I, Estivill X, et al A highly expressed miR-101 isomiR is a functional silencing small RNA. BMC Genomics. 2013;14: 104–104. 10.1186/1471-2164-14-104 23414127 PMC3751341

[72] Carmi S, Borukhov I, Levanon EY. Identification of Widespread Ultra-Edited Human RNAs. Maizels N, editor. PLoS Genet. 2011;7: e1002317 10.1371/journal.pgen.1002317 22028664 PMC3197674

[73] Bass BL. RNA editing by adenosine deaminases that act on RNA. Annu Rev Biochem. 2002;71: 817–846. 10.1146/annurev.biochem.71.110601.135501 12045112 PMC1823043

[74] Chen CF, Ridzon DA, Broomer AJ, Zhou ZH, Lee DH, Nguyen JT, et al Real-time quantification of microRNAs by stem-loop RT-PCR. Nucleic Acids Res. 2005;33 10.1093/nar/gni178 PMC1292995 16314309

[75] Rapaport F, Khanin R, Liang Y, Pirun M, Krek A, Zumbo P, et al Comprehensive evaluation of differential gene expression analysis methods for RNA-seq data. Genome Biol. 2013;14 10.1186/gb-2013-14-9-r95 PMC4054597 24020486

[76] Anders S, Huber W. Differential expression analysis for sequence count data. Genome Biol. 2010;11: R106 10.1186/gb-2010-11-10-r106 20979621 PMC3218662

[77] Abdellatif M. The Role of MicroRNA-133 in Cardiac Hypertrophy Uncovered. Circulation Research. 2010;106: 16 10.1161/CIRCRESAHA.109.212183 20056941 PMC2838710

[78] Yin VP, Lepilina A, Smith A, Poss KD. Regulation of zebrafish heart regeneration by miR-133. Developmental Biology. 2012;365: 319–327. 10.1016/j.ydbio.2012.02.018 22374218 PMC3342384

[79] Gurha P, Abreu-Goodger C, Wang T, Ramirez MO, Drumond AL, van Dongen S, et al Targeted Deletion of MicroRNA-22 Promotes Stress-Induced Cardiac Dilation and Contractile Dysfunction. Circulation. 2012;125: 2751–2761. 10.1161/CIRCULATIONAHA.111.044354 22570371 PMC3503489

[80] Huang ZP, Chen J, Seok HY, Zhang Z, Kataoka M, Hu X, et al MicroRNA-22 Regulates Cardiac Hypertrophy and Remodeling in Response to Stress. Circulation Research. 2013;112: 1234–1243. 10.1161/CIRCRESAHA.112.300682 23524588 PMC3720677

[81] Han M, Yang Z, Sayed D, He M, Gao S, Lin L, et al GATA4 expression is primarily regulated via a miR-26b-dependent post-transcriptional mechanism during cardiac hypertrophy. Cardiovasc Res. 2012;93: 645–654. 10.1093/cvr/cvs001 22219180 PMC3291090

[82] Wong SSY, Ritner C, Ramachandran S, Aurigui J, Pitt C, Chandra P, et al miR-125b Promotes Early Germ Layer Specification through Lin28/let-7d and Preferential Differentiation of Mesoderm in Human Embryonic Stem Cells. Emanueli C, editor. PLoS ONE. Public Library of Science; 2012;7: e36121–12. 10.1371/journal.pone.0036121 22545159 PMC3335794

[83] Wang X, Ha T, Zou J, Ren D, Liu L, Zhang X, et al MicroRNA-125b protects against myocardial ischaemia/reperfusion injury via targeting p53-mediated apoptotic signalling and TRAF6. Cardiovasc Res. 2014;102: 385–395. 10.1093/cvr/cvu044 24576954 PMC4030511

[84] van Rooij E, Quiat D, Johnson BA, Sutherland LB, Qi X, Richardson JA, et al A Family of microRNAs Encoded by Myosin Genes Governs Myosin Expression and Muscle Performance. Dev Cell. Elsevier Ltd; 2009;17: 662–673. 10.1016/j.devcel.2009.10.013 19922871 PMC2796371

[85] Oliveira-Carvalho V, Carvalho VO, Bocchi EA. The Emerging Role of miR-208a in the Heart. DNA Cell Biol. 2013;32: 8–12. 10.1089/dna.2012.1787 23121236

[86] Vacchi-Suzzi C, Hahne F, Scheubel P, Marcellin M, Dubost V, Westphal M, et al Heart Structure-Specific Transcriptomic Atlas Reveals Conserved microRNA-mRNA Interactions. Hosoda T, editor. PLoS ONE. 2013;8: e52442–13. 10.1371/journal.pone.0052442 23300973 PMC3534709

[87] Liu X, Xiao J, Zhu H, Wei X, Platt C, Damilano F, et al miR-222 Is Necessary for Exercise-Induced Cardiac Growth and Protects against Pathological Cardiac Remodeling. CMET. Elsevier; 2015;21: 584–595. 10.1016/j.cmet.2015.02.014 PMC4393846 25863248

[88] Chistiakov DA, Sobenin IA, Orekhov AN, Bobryshev YV. Human miR-221/222 in Physiological and Atherosclerotic Vascular Remodeling. BioMed research international. 2015;2015: 1–18. 10.1155/2015/354517 PMC4499635 26221589

[89] Vacchi-Suzzi C, Bauer Y, Berridge BR, Bongiovanni S, Gerrish K, Hamadeh HK, et al Perturbation of microRNAs in Rat Heart during Chronic Doxorubicin Treatment. Martelli F, editor. PLoS ONE. 2012;7: e40395–11. 10.1371/journal.pone.0040395 22859947 PMC3409211

[90] McGahon MK, Yarham JM, Daly A, Guduric-Fuchs J, Ferguson LJ, Simpson DA, et al Distinctive Profile of IsomiR Expression and Novel MicroRNAs in Rat Heart Left Ventricle. Martelli F, editor. PLoS ONE. 2013;8: e65809 10.1371/journal.pone.0065809 23799049 PMC3683050

